# Using human urinary extracellular vesicles to study physiological and pathophysiological states and regulation of the sodium chloride cotransporter 

**DOI:** 10.3389/fendo.2022.981317

**Published:** 2022-08-29

**Authors:** Aihua Wu, Martin J. Wolley, Robert A. Fenton, Michael Stowasser

**Affiliations:** ^1^ Endocrine Hypertension Research Centre, University of Queensland Diamantina Institute, Greenslopes and Princess Alexandra Hospitals, Brisbane, QLD, Australia; ^2^ Department of Nephrology, Royal Brisbane and Women’s Hospital, Brisbane, QLD, Australia; ^3^ Department of Biomedicine, Aarhus University, Aarhus, Denmark

**Keywords:** urinary extracellular vesicles, sodium chloride cotransporter, potassium, aldosterone, hypertension, primary aldosteronism

## Abstract

The thiazide-sensitive sodium chloride cotransporter (NCC), expressed in the renal distal convoluted tubule, plays a major role in Na^+^, Cl^-^ and K^+^ homeostasis and blood pressure as exemplified by the symptoms of patients with non-functional NCC and Gitelman syndrome. NCC activity is modulated by a variety of hormones, but is also influenced by the extracellular K^+^ concentration. The putative “renal-K^+^ switch” mechanism is a relatively cohesive model that links dietary K^+^ intake to NCC activity, and may offer new targets for blood pressure control. However, a remaining hurdle for full acceptance of this model is the lack of human data to confirm molecular findings from animal models. Extracellular vesicles (EVs) have attracted attention from the scientific community due to their potential roles in intercellular communication, disease pathogenesis, drug delivery and as possible reservoirs of biomarkers. Urinary EVs (uEVs) are an excellent sample source for the study of physiology and pathology of renal, urothelial and prostate tissues, but the diverse origins of uEVs and their dynamic molecular composition present both methodological and data interpretation challenges. This review provides a brief overview of the state-of-the-art, challenges and knowledge gaps in current uEV-based analyses, with a focus on the application of uEVs to study the “renal-K^+^ switch” and NCC regulation. We also provide recommendations regarding biospecimen handling, processing and reporting requirements to improve experimental reproducibility and interoperability towards the realisation of the potential of uEV-derived biomarkers in hypertension and clinical practice.

## Introduction

Hypertension (using the cut-off of >139/89 mmHg) affects up to 40% of adults worldwide, and is a major risk factor for stroke, coronary heart disease, heart failure, chronic kidney disease and development of severe coronavirus infection complications (1–4). Excessive dietary sodium (Na^+^) intake is generally accepted to play a role in the development of hypertension (5). However growing evidence now spotlights an inverse association between dietary potassium (K^+^) and blood pressure (BP) (6–8). A recent population-wide salt substitution initiative which replaced regular salt with a K^+^- enriched salt substitute in Peru reported an average reduction of 1.29 in systolic BP and 0.76 in diastolic BP, and an over 50% reduction in hypertension incidence in normotensive people (9). The K^+^-enriched salt substitution trial in populations with history of stroke or hypertension demonstrated to lower the rates of stroke, major cardiovascular events and death, and participants demonstrated a mean reduction of 3.34 mmHg in systolic BP after five years (10). These observations indicate that the impact of dietary K^+^ may be even higher than that of dietary Na^+^ in regulation of body fluid volume and BP maintenance.

Renal Na^+^ handling has a profound effect on body fluid and BP maintenance as exemplified by the use of diuretic medications to treat states of volume expansion or hypertension. The thiazide-sensitive NaCl cotransporter (NCC, encoded by *SLC12A3*) plays a key role in the regulation of Na^+^, Cl^-^ and K^+^ balance and BP, as underscored by Gitelman syndrome (loss-of-function mutation in *SLC12A3*) resulting in normal to low blood pressure, hypokalaemia and metabolic alkalosis, combined with significant hypomagnesaemia and hypocalciuria. Activation of the renin-angiotensin-aldosterone system (RAAS) was initially thought to be a primary driver of NCC activity, but this system could not easily explain the observations that NCC activity decreased during high aldosterone states subsequent to high dietary K^+^ intake (11). This conundrum was recently solved when it was demonstrated that a reduction in plasma K^+^ (for example due to reduced dietary K^+^ intake or prolonged aldosterone stimulation) increased NCC activity (12, 13). This mechanism may contribute to the volume expansion and hypertension associated with conditions associated with aldosterone excess (12, 13). The effects on NCC are dependent on a variety of K^+^ mediated alterations in cellular signaling, which together orchestrate the putative “renal-K^+^ switch”, a mechanism which may offer new targets for pharmaceutical or dietary manipulation in health and disease. However, although the renal-K^+^ switch has been robustly tested in various animal models, a hurdle that still exists is the lack of data to confirm the existence and relevance of this mechanism in humans.

Extracellular vesicles (EVs) are small vesicles derived from their cells of origin and have been detected in blood, urine and other biofluids. Urine is a favorable specimen for biomarker discovery and is used to diagnose and monitor kidney diseases. Proteome analyses reveal that EVs isolated from urine may represent a more targeted approach for biomarker discovery than unfractionated urine (14–20). EVs originating from cells lining the urinary tract contain molecules derived from glomerular, tubular and bladder cells (21), and EVs released from renal tubules carry protein channels from different segments that are responsible for Na^+^ and water reabsorption under hormonal regulation (22). Therefore, analysis of urinary EVs can serve as a non-invasive novel approach to study physiological and pathophysiological states and regulation of NCC in humans.

## Urinary extracellular vesicles

Urinary extracellular vesicles (uEVs) are a heterogenous group of nanosized membrane vesicles excreted by cells lining the urinary tract (21) that function as a carrier of information (e.g. proteins (23), lipids (24) and nucleic acids (25–27)) for cell-to-cell communication and intercellular exchange. The uEV proteome and transcriptome contains multiple disease-associated proteins and transcripts (18, 28), indicating that uEVs are a non-invasive source of potential molecular biomarkers to mirror molecular processes as well as physiological and pathological conditions in the kidney and other urinary tract tissues (16, 21, 22). Although uEVs are an attractive research tool, their diverse origins and dynamic molecular composition present an enormous methodological challenge. This review aims to give a brief overview of the state-of-the-art, challenges and knowledge gaps in current uEV-based analyses, with a focus on the application of uEVs to study the “renal-K^+^ switch” and NCC regulation. We also provide recommendations regarding biospecimen handling, processing and reporting requirements to improve experimental reproducibility and interoperability towards the realization of the potential of uEV-derived biomarkers in hypertension and clinical practice.

### Biology of EVs

**Figure 1:**
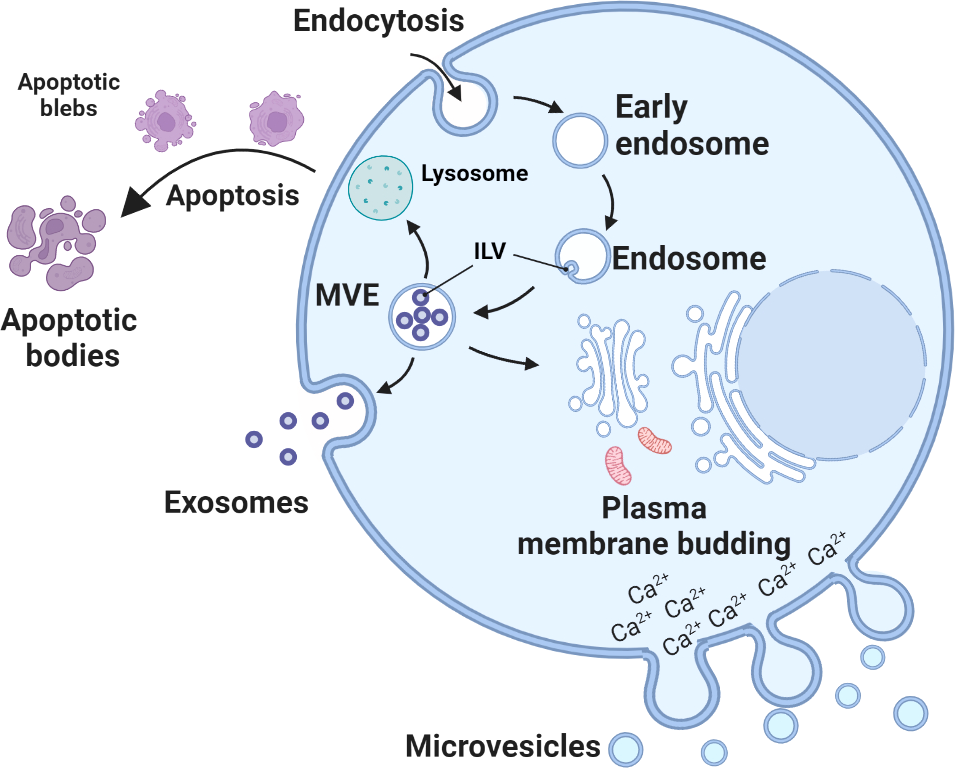
Biogenesis of urinary extracellular vesicles (uEVs). Exosome biogenesis starts from inward budding of the plasma membrane (endocytosis) and eventual formation of early endosomes. The membranes of early endosomes invaginate and bud into surrounding luminal space with cytoplasmic content to form intraluminal vesicles (ILVs) (29). Late endosomal structures containing ILVs are known as multivesicular endosomes (MVEs), which are eventually transported to the trans-Golgi network for endosome recycling, delivered to lysosomes for degradation of all carried material, or fuse with the plasma membrane and release exosomes outside the cell (30). Microvesicles arises through outward budding and fission of plasma membrane and is the result of dynamic interplay between phospholipid redistribution and cytoskeletal protein contraction (31, 32). Apoptotic bodies are formed during apoptosis. Apoptosis progresses through several stages, first nuclear chromatin condensation, then nuclear splitting and the frequent appearance of micronuclei, then membrane blebbing and finally splitting of the cellular content into distinct membrane-enclosed vesicles, termed apoptotic bodies (33, 34).

EVs consist of exosomes, microvesicles and apoptotic bodies (**Figure 1**). The biogenesis of exosomes and microvesicles both involve membrane trafficking processes. Exosomes are generated as intraluminal vesicles in the lumen of multivesicular endosomes by inward budding of the endosomal membrane during the formation and maturation of multivesicular endosomes (MVEs). Their biogenesis pathways are intermediated within the endosomal system and released outside the cells upon fusion of MVEs with the cell surface for intercellular communication (35, 36). In contrast, accumulation of calcium-dependent enzymes and changes in the polarity of membrane phospholipids (37) cause outward budding and fission of the plasma membrane and the subsequent release of microvesicles into the extracellular space (38). Apoptotic bodies are formed during apoptosis when cells undergo characteristic outward blebbing caused by breaks in the cytoskeleton (33, 34). Once released by the cell, microvesicles and exosomes exhibit overlapping size and composition, which makes it difficult to determine their origin.

### Origin of uEVs

**Figure 2:**
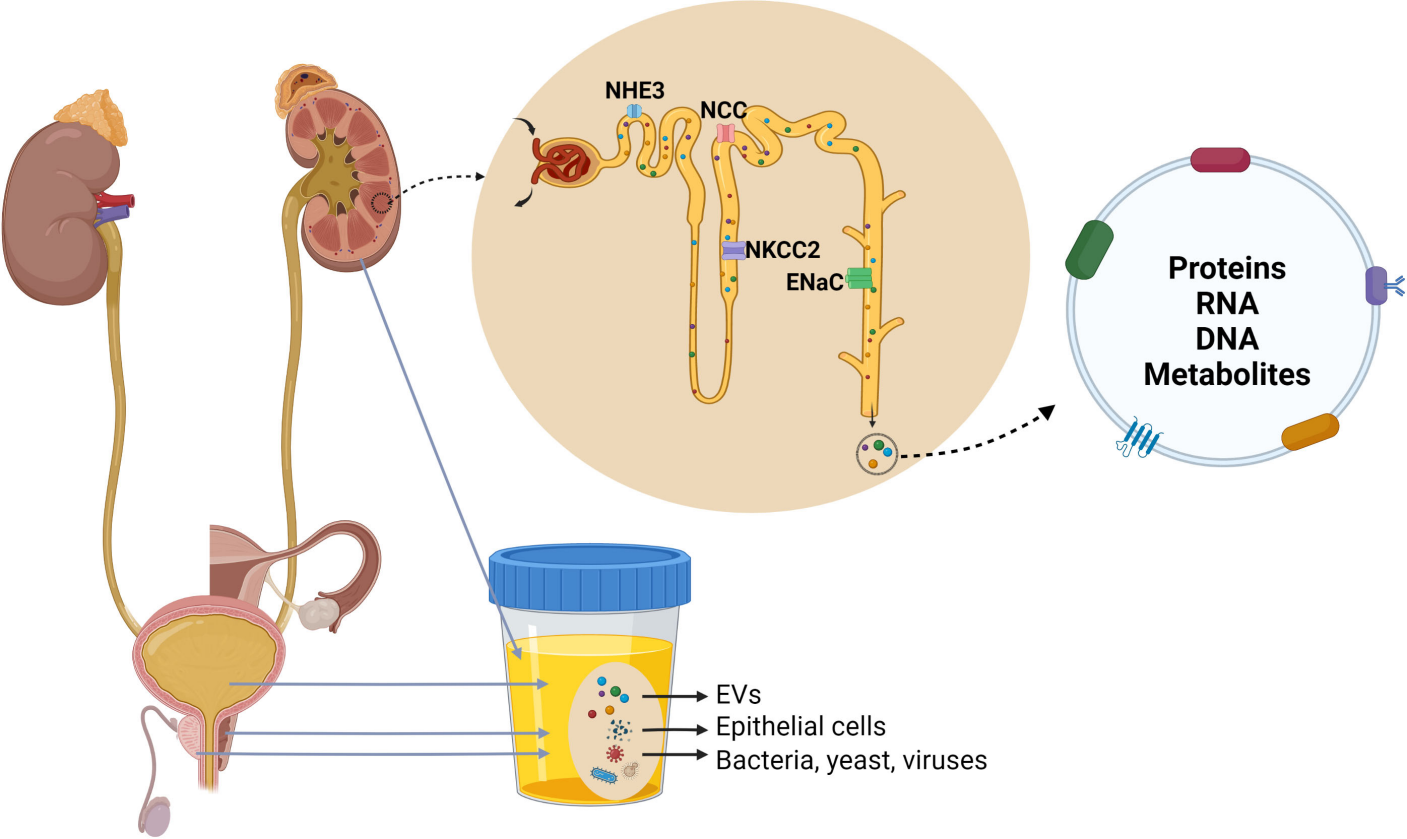
Origin and composition of urinary extracellular vesicles (uEVs). uEVs are generally considered to originate from several parts of the urogenital tract, including the kidneys, bladder, prostate in males and utero-vaginal tract in females. Urine can also contain a small quantity of EVs derived from other organs, epithelial cells, bacteria, yeast and viruses. uEVs carry proteins, nucleic acids, lipids and metabolites, and uEVs originating from cell lining nephron lumen contain marker proteins of nephron segments.

uEVs originate from several parts of the urogenital tract, including the kidneys, bladder, prostate in males and utero-vaginal tract in females **(** **Figure 2** **)** (14, 39), and can be differentiated by characteristic proteins (14, 39, 40). However, the relative contributions of each part of the urogenital tract to the total population of uEVs has not been determined. Non-urinary tract material (proteins, microRNA etc.) or systemic EVs can also be detected in urine (41, 42), suggesting that EVs can cross the glomerular filtration barrier and basement membrane of the kidney. How this occurs is unclear, but recent studies in transgenic mice have emphasized that under physiological conditions the majority of uEVs are derived from the kidney with limited contribution of EVs from the circulation (43). In contrast, in pathophysiological states this contribution may be different. For example, during lung cancer, uEVs have been reported to contain tumour-specific proteins (44). Recent studies have demonstrated that cancer-cell derived exosomes can cause blood-brain-barrier leakiness by modulating actin dynamics of recipient endothelial cells, resulting in the breakdown of tight junctions, higher vascular permeability and metastasis (45–47). Exosome-mediated dysfunction of glomerular filtration has also been suggested, with glomerular endothelial cell-derived exosomes in a high glucose environment triggering epithelial-mesenchymal transition and dysfunction of podocytes (48). Therefore, it is likely that various pathophysiological states impair the glomerular filtration barrier allowing passage of systemic EVs into the urinary space. EVs in the urine can also be derived from residing immune cells and bacteria (49, 50). As urinary immune effectors, uEVs from healthy individuals are enriched in proteins involved in host defence and immunity with bacteriostatic/bactericidal functions, and proteins that bind to bacterial surface molecules (51).

### Molecular composition

uEVs carry proteins, nucleic acids, lipids and metabolites. However, because uEVs are a population of vesicles commonly obtained following multiple steps, the actual composition of each subtype of uEVs is unclear.

The protein composition of uEVs was originally studied in the context of the feasibility of AQP2 detection in low-density membrane vesicles isolated from human urine by ultracentrifugation (52, 53). Thereafter, several major Na^+^ transporters were identified in urine low density membrane fractions, such as Na/H exchanger 3(NHE3), Na-K-2Cl cotransporter 2 (NKCC2) and NCC (54). By 2011, studies using mass spectrometry techniques identified more than 1000 proteins in human uEVs (14, 39, 55, 56), including biomarkers for the renal and urogenital system in pathological conditions (57–60). Improvements in mass spectrometric techniques have expanded the number of proteins identified in uEVs to over 3000 (61, 62), enabling deeper analysis of EV biology and identification of additional biomarker candidates (63–66).

Various RNA species are present in uEVs, and include protein coding transcripts (mRNAs) and a prominent population of small non-coding RNAs. mRNAs in uEVs derive from all regions of the nephron and could facilitate the examination of kidney cellular transcriptional changes in health and disease (67). The small non-coding RNAs in uEVs include microRNAs (miRNAs), small nuclear RNAs (snRNAs), small nucleolar RNAs (snoRNAs), transfer RNAs (tRNAs) and long non-coding RNAs (lncRNAs) (68–71), which play an essential role in intracellular communication by transferring genetic information. Additionally, uEVs contain over 10,000 of the ~20,000 known protein-coding genes and ribosomal RNA (rRNA) transcripts, which is more abundant compared to EVs from other human biofluids (71, 72). Similar to kidney tissue, uEVs have an RNA integrity profile with prominent 18S and 28S rRNA peaks (67). This finding suggests that the EV structure protects the RNAs from degradation by ribonucleases during urine passage, thus increasing the potential of using uEVs as a source of reliable RNA-based biomarkers. However, the presence of 18S and 28S rRNA in uEVs is not consistent (69, 73, 74). It remains unclear whether DNA is present in the lumen of uEVs, but uEVs with and without DNase treatment showed similar trends in read distribution of nucleic acid cargo following deep sequencing (71).

Lipids and metabolites are relatively less investigated uEV components. However, in human uEVs over 100 lipid species have been detected by mass spectrometry, with cholesterol the most abundant lipid species, followed by phosphatidyl serine (75). Compared to EVs from other human biofluids, only in uEVs, all phosphatidylethanolamine species were identified as phosphatidylethanolamine-ether lipid species (75). The amount of ether lipids in EVs can be regulated, and higher levels of ether lipids in EVs have been associated with greater EV release and altered EV protein composition (76). A study that profiled metabolites in uEVs identified six main categories: organic acids and their derivatives, nucleotides, sugars and derivatives, carnitines, vitamin B/related metabolites, and amines. Among the most abundant metabolites were ornithine, D-ribose 5-phosphate, L-cystathionine, alanine and serine (77).

## Current technologies for studying uEVs

The general steps for studying uEVs include pre-analytical urine handling, uEV isolation, uEV characterisation and normalisation, and downstream analyses of uEV content. Careful consideration of the applied technologies and approaches used at each step are necessary as these aspects are major source of data variability and can limit data reproducibility (16, 78–80).

### Pre-analytical urine handling

Pre-analytical urine handling is a critical source of variability as preservation and storage methods have a major impact on outcomes. Important steps in urine handling include urine collection (e.g. time of voiding, use of protease inhibitors), processing and storage (e.g. immediate freezing at minus twenty or eighty degrees, short-term storage at 4°C, uEV isolation before storage), and urine handling after thawing (e.g. rapid or slow thawing, extensive vortexing). Huebner and colleagues summarized and compared currently applied methods for urine collection and storage for uEV utilization (16). Their studies concluded that the addition of protease inhibitors and preservatives such as phenylmethanesulfonyl fluoride, leupeptin, and sodium azide, long-term storage at -80°C, and extensive vortexing of urine after thawing provide the best quality uEVs for subsequent analyses (**Table 1**). Technical details of each step should be standardized for clinical and research purpose because data can be profoundly influenced by these pre-analytical variables.

**Table 1:** Important steps of pre-analytical urine handling.

Steps	Impact factors	Comments
Urine collection	Spot urine/timed urine	Spot urine: first or second morning urine have similar EV contents and they are suggested to be used interchangeably for experimental research purposes (80). However, specific uEV biomarkers may significantly altered between the two collections and several renal functions have circadian rhythms (81, 82). The choice between first and second morning urine depends on the pathology being investigated.
Addition of inhibitors/enzymes	With/without protease/phosphatase inhibitors	Some key proteins often detected in uEVs degrade without used of protease inhibitors (83). Immediately addition is necessary.
	With/without DNase	No large differences when comparing the read distribution of the uEV inner nucleic acid (67), but no addition of DNase increases intergenic and intronic reads (84). If needed, immediate addition is necessary.
Storage	4°C/-20°C/-80°C	Use fresh uEV isolates (4°C) for best results of morphological characterisation by electron microscopy (84); -20°C results in >50% loss of EVs, -80°C causes 14% EV loss (80); -80°C offers a more stable condition for long-term use (83).
Defrosting	Room temperature/4°C overnight/thawing under running water	Unknown effects of thawing method on uEVs, but likely needs attention when studying heat liable molecules
Extensive vortexing after thawing	Yes/no	Over 87% recovery of EVs with extensive vortexing after thawing (83)

### uEV isolation

Ultracentrifugation was the first method used to reproducibly isolate uEVs (14). However, the resulting uEV pellet includes contamination with highly abundant urinary proteins such as Tamm-Horsfall protein (THP, also known as uromodulin) and albumin in pathological states. THP originates from a glycosylphosphatidylinositol-linked protein present in the apical plasma membrane of the thick ascending limb of the Henle loop, and is excreted into urine by proteolytic cleavage (85). THP forms large networks of fibre that can constrain uEVs in the urine and interfere with fractioning procedures (86). Hence, techniques to remove or eliminate THP in urine before uEV isolation are required to enhance final yield. Progressive ultracentrifugation is the most commonly used method (14). Initially, low speed centrifugation is used to remove cells and debris, followed by incubation of the resuspended low-density membrane pellet with reducing agents and subsequent repeated ultracentrifugation. This procedure denatures the zona pellucida domains in THP, thus inhibiting aggregation and allowing THP to be removed in the supernatant. Reducing agents, such as dithiothreitol (DTT) can depolymerize THP at 37°C by breaking disulfide bridges between individual THP monomers (87–90), which effectively eliminates polymeric entrapment of uEVs. However, DTT may also cause remodeling of the exosomal proteins, therefore hampering relevant functional studies and reducing the potential for using affected proteins as biomarkers (87). The detergent 3-[(3-cholamidopropyl) dimethylammonio]-1-propanesulfonic (CHAPS) has the capability of solubilising THP while at the same time preserving the conformation and the enzymatic activity of proteins contained within uEVs (88, 91), hence it can be considered as an optimised substitute of DTT if remaining physiological activity in the sample is of importance. Unfortunately, both uEV treatments with DTT and CHAPS did not completely remove THP or other abundant urinary proteins (92). Therefore modifications such as the use of double-cushioning (93), use of heavy water and a sucrose gradient in ultracentrifugation (94), or ultracentrifugation followed by size-exclusion chromatography (95) have been introduced to improve uEV purity. However, the long and laborious processing times and the requirement of expensive equipment have rendered them less attractive for clinical application.

New methodologies used for uEV isolation include EV filtration techniques, uEV precipitation, hydrostatic dialysis, acoustic trapping and immunocapture (**Table 2**). These methods have shown varying degrees of improved efficiency and yield, but further evaluation is required to determine optimal isolation methods for different analytical purposes (e.g., measurement of proteins, RNAs or lipids). In addition, the patient’s clinical condition should be considered in the isolation approach because, depending on the disease state involved, urine may contain various amounts of albumin, bacteria, erythrocytes, lymphocytes or other potentially interfering substances. For example, in glomerular disease, albumin and other proteins that leak into urine can bind to the surface of EVs or form a protein complex (101, 102). Therefore, isolation methods that can reduce albumin and other proteins in uEV isolates (e.g. ultracentrifugation followed by size-exclusion chromatography, the use of sucrose or other density gradients or hydrostatic filtration dialysis) are recommended (101, 103).

**Table 2:** Methodologies used for uEV isolation.

Technique	Isolation method	Advantages	Disadvantages
Ultracentrifugation	Progressive ultracentrifugation (14)	Reproducible results; high yield of intact proteins and nucleic acids	5-7 h to process single sample; contamination by highly abundant proteins; expensive equipment.
	Double-cushion ultracentrifugation (93)	Less contamination of highly abundant proteins; reproducible results	Long processing time; tedious separation techniques; expensive equipment
	Sucrose gradient ultracentrifugation (94)		
	Ultracentrifugation-size exclusion chromatography (95)		
Filtration	Nanomembrane filtration (96)	Shorter processing time (0.5-2 h); many samples can be processed at one time; relatively inexpensive; Can be used in a clinical setting	Possible clogging of membrane; sample loss; contamination by highly abundant proteins
	Micromembrane filtration (97)		
Precipitation	Precipitation by ExoQuick-TC (15)	Shorter processing time (0.5-2 h); Yields intact RNA; Relatively inexpensive; can be used in a clinical setting.	Low purity of protein; Modified protocol.
Hydrostatic dialysis	Hydrostatic filtration dialysis (73)	Low cost, simple system, efficient pre-processing and concentration for biobanking purposes; suitable to any downstream analyses; patients can be the end-users	Protein purity is not as good as ultracentrifugation, but acceptable; contain THP contamination; Comparing to ultracentrifugation, large vesicle fraction (>500 nm) was underrepresented, low proportion of small EVs (60-140 nm) and more very small size EV-like participles (<40nm) (72).
Acoustic trapping	Polystyrene beads model (98)	Rapid, automated, low-volume compatible, robust; no impact on the integrity or miRNA content of trapped vesicles	Amplifier used to drive the piezo may limit the possibilities for device parallelization (99)
Immunocapture	Antibody-based affinity capture on magnetic beads (100)	Less expensive equipment, less expertise, purer uEV fractions	Capture proteins displayed on the outer surface of uEVs

### uEV characterisation

EV characterisation is not straightforward, because non-EV entities, such as argonaut 2 protein complex and lipoproteins also contain components that are present in EVs (104, 105), and the commonly used protocols and commercial kits that claim high quality EV or exosome isolation or purification cannot fully separate EVs from non-EV entities (106). In addition, variability in experimental systems, investigator experience and the instrumentation used contribute to the heterogeneity of protocols and composition of recovered EVs, leading to difficulties in interpretation of results. The International Society for Extracellular Vesicles has recently provided updated criteria to guide researchers in EV characterisation for the purposes of single EV characterisation, quantification, and global characterisation (107). Detailed requirements of EV characterisation are summarised in **Table 3**.

**Table 3:** EV characterisation methods (107).

Characterisation type	Details	Requirement
Quantification	Volume of fluid, and/or cell number, and/or tissue mass used to isolate EVs	Mandatory
	Global quantification by at least two methods: protein amount, particle number, lipid amount, expressed per volume of initial fluid or number of producing cells/mass of tissue	Mandatory
Global characterisation	Ratio of the 2 quantification figures	Mandatory
	Transmembrane or GPI-anchored protein localised in cells at plasma membrane or endosomes: e.g., tetraspanins (CD9, CD63, CD81), integrins or cell adhesion molecules, growth factor receptors, heterotrimeric G proteins, phosphatidylserine-binding MFGE8/lactadherin	Mandatory
	Cytosolic protein with membrane-binding or association capacity: e.g., endosome or membrane-binding proteins (tumor susceptibility gene 101 protein (TSG101), annexins, Rabs, signal transduction or scaffolding proteins (synthenin)	Mandatory
	Assessment of presence/absence of expected contaminants: e.g., endoplasmic reticulum specific proteins (Grp94, calnexin, Golgi, mitochondria), nucleus specific (histones), Argonaute/RISC complex	Mandatory
	Presence of proteins associated with compartments other than plasma membrane or endosomes	Mandatory if applicable
	Presence of soluble secreted proteins and their likely transmembrane ligands	Mandatory if applicable
	Topology of the relevant functional components	Encouraged
Single EV characterisation	Image of single EVs by wide-field and close-up: e.g., electron microscopy, scanning probe microscopy, super-resolution fluorescence microscopy.	Mandatory
	Non-image-based method analysing large numbers of single EVs: NTA, TRPS, FCS, high-resolution flow cytometry, multi-angle light-scattering, Raman spectroscopy, etc.	Mandatory

Unfortunately, there is no single technique that can fulfill the requirements for describing EV morphology, size, number and content. Current techniques used for EV morphological characterisation include transmission electron microscopy (TEM), cryogenic electron microscopy (cryo-EM), atomic force microscopy (AFM) and super resolution fluorescence microscopy, among which TEM is the most commonly used. Because these techniques provide different information about EV structure and size distribution, they are not necessarily interchangeable or capable of providing images of comparable quality. Techniques used to measure uEV size distribution and count include nanoparticle tracking analysis (NTA) and tunable resistive pulse sensing (TRPS). On the basis of Brownian motion, NTA provides uEV particle size distribution and concentration within a specific detection range, however it cannot exclude non-EV entities and therefore NTA-based particle count may be overestimated. TRPS is a relatively more accurate method that measures particle size, number and surface charge (90). Complementing these approaches, assessment of the presence of EV-enriched/specific molecules (e.g. EV housekeeping genes/proteins) and absence of potential contaminants by standard methodologies are often utilised to describe EV content for the purpose of global characterisation. For example, uEV protein content can be measured by bulk analysis (western blotting, ELISA) or high-throughput flow cytometry for single EV surface protein analysis. Techniques currently used for uEV characterisation are listed in **Table 4**.

**Table 4:** Current techniques for uEV characterisation.

Characterisation	Technique	Information giving	Pros and cons
Morphology	Transmission electron microscopy (TEM)	Images of a heterogeneous group of EVs of different sizes and shapes for sample purity; TEM also shows EV heterogeneity by different staining densities to highlight morphological characteristics and surface features.	Pros: easier and more accessible than cryo-EM; commonly used for EV morphology. Cons: expensive and time consuming; must have a very thin layer.
	Cryogenic electron microscopy (cryo-EM)	Cryo-EM shows the lipid-bilayer and all particles in a given volume can be imaged, not just those that adhere to a surface (the grid) (108, 109).	Pros: preserves EV size better than the dehydrating conditions used to fix samples for TEM and may be more quantitative. Cons: costly equipment that requires specialized staff to setup.
	Atomic force microscopy (AFM)	Visualisation of uEVs with sub-nanometer resolution in three dimensions in atmospheric or submerged conditions (110).	Pros: samples do not require any special treatments that would irreversibly change or damage the sample; most AFM modes work well in ambient air or a liquid environment. Cons: can only obtain surface information from samples; also limited by the single scan image size and the relatively slow scanning speed.
	Super resolution fluorescence microscopy	Direct visualization of fluorescently labelled molecules within vesicles with 20 nm resolution, revealing the biomarker distribution and expression levels on single vesicles (111–113).	Pros: provides better spatial resolution for observing exosomes and enables intracellular tracking of exosomes. Cons: special fluorophores required; phototoxicity associated with multiple imaging/quenching cycles; imaging close to coverslip.
Size distribution and counts	Nanoparticle tracking analysis (NTA)	Particle size distribution and particle concentration within a range.	Pros: accessible and commonly used for EV morphology. Cons: cannot exclude non-EV entities; particle count may be overestimated; may generate biased results due to calibrators in use; different software generates different absolute particle count.
	Tunable resistive pulse sensing (TRPS)	EV particle size distribution, particle number and surface charge (90)	Pros: rapid, convenient, accurate and reproducible. Cons: discrepancy in count numbers between TRPS and NTA.
EV content	Western blotting/ELISA	Specific uEV content	Pros: easy and accessible; widely used for analysis and validation of one or a few target proteins. Cons: requires validated antibodies.
	Flow cytometry	Single EV surface protein	Pros: bead-based commercial kit are available (114). Cons: requires experienced staff to setup instrument for sufficient resolution (115).
	Liquid chromatography-tandem mass spectrometry (LC-MS/MS)	Protein profile within uEVs (116)	Pros: precise, rapid and sensitive; requires small sample size to produce data that can reach high statistical power. Cons: expensive in terms of capital and running costs; needs a skilled technician.
	RNA-sequencing	Transcriptome of uEVs (117)	Pros: sequencing of small RNAs and targeted or capture sequencing of longer RNAs has proved to be successful. Cons: total RNA sequencing is limited by short fragment length, low number of quantified genes or a high level of ribosomal RNA contamination.
	Ultra-performance liquid chromatography coupled to mass spectrometry (UPLC–MS)	Lipids and metabolites of uEVs (77, 118).	Pros: fast analysis of small molecular weight samples. Cons: problems associated with dangerous organic solvents in use which are toxic and injurious to the environment.

### Normalisation

One of the greatest challenges in uEV-based research is the requirement for normalisation of uEV contents between samples or subjects, especially in clinical studies where spot urine is the most frequently used and simplest collection method. Current normalisation approaches, each with both benefits and limitations, include comparing uEV contents to THP abundance, urine creatinine concentration, known EV-enriched proteins, uEV concentration or uEV numbers. THP abundance highly correlates with EV markers such as ALG-2-interacting protein X (ALIX) and Tumor Susceptibility Gene 101 Protein (TSG101) (87), but the association between THP aggregation (polymerization) and the ionic strength and pH of urine needs to be taken into account (119). In addition, THP is less suitable for normalisation when DTT or other reducing reagents are used during uEV isolation.

Urine creatinine is widely used to normalize analytes (e.g. urine protein-to-creatinine ratio) in spot urine samples in routine clinical practice (120), but its use in uEV normalisation is less common (121–126). A recent study quantifying urine particle in dilute and concentrated urines randomly obtained from healthy volunteers and subjects with kidney disease demonstrated a strong correlation between uEV concentration and urine creatinine (127), indicating urine creatinine can potentially be used to normalize spot urines. However, normalisation for creatinine concentration in spot urines does not address the influence of variable uEV recovery/sedimentation rates during progressive ultracentrifugation or other isolation approaches (128).

The abundance of EV-enriched proteins and uEV concentration are considered relatively stable (129). As such, EV-enriched proteins such as tetraspanins surface markers (CD9, CD63) and proteins of exosomal biogenesis (ALIX, TSG101) are commonly used to characterize uEVs and for normalisation/correction of protein abundances in uEVs. However, some proteins may not be generally applicable for all urine samples. Blijdorp et al. reported that the commonly used uEV markers CD9 and CD63 are differentially expressed throughout the urogenital system, and therefore use of CD9 or CD63 as a normalizer can affect final outcomes (127). Importantly, for unbiased uEV characterisation methods using mass spectrometry or real-time quantitative polymerase chain reaction, no reliable normalisation or housekeeping target is currently available.

The uEV concentration depends not only on the isolation method used, but also the uEV production and excretion rate (secretion minus possible uptake) and the overall urine concentration at the sampling time point. uEV excretion has showed a circadian pattern that is independent of sex, fed-fasting/hydration status and kidney injury, and this circadian variation can be normalised by uEV particle number and its marker protein TSG101 (130). A more recent study demonstrated nephron mass determines uEV excretion rate, but nephrectomy reduces uEV excretion less than expected based on nephron loss due to compensatory hypertrophy (131). The findings imply that proxies for nephron mass including kidney function (e.g., eGFR and creatinine clearance), total kidney volume and kidney weight should also be included in uEV-based studies for inter-individual comparisons, but these indicators may not suit for studies in nephrectomy patients. The same authors furthermore identified a sex difference in uEV excretion that is likely due to greater nephron mass in men, which implies that when the excretion of a uEV biomarker is studied in males and females, the results should be corrected for uEV excretion rate to avoid false estimation of biomarker levels. Nonetheless, a validated normalisation method, or more specifically a normalisation variable, is urgently required to substitute for time in analysing the relative excretion rate of uEV proteins (87).

## Reliability of uEVs in studying physiological and pathophysiological processes in kidney

Measurement of uEV protein abundances is frequently used to reflect ongoing changes in the kidney or identify biomarkers for specific diseases. For example, uEVs have been used for quantitative assessment of the levels of different proteins in the setting of patients with familial hyperkalaemic hypertension (132), Bartter syndrome (133), Gitelman syndrome (133, 134), primary aldosteronism (135–139), Cushing’s syndrome (140), polycystic kidney disease (49) and nephrogenic diabetes insipidus (123). However, matched kidney and uEV samples isolated from patients undergoing nephrectomy demonstrated no correlations in the abundance of nine proteins between the two sources (141). Although there were technical limitations, such as the correlations being based on a limited number of proteins and a significant period of time between urine and tissue collection, this study raised doubt as to whether changes in uEV protein content faithfully reflect changes within kidney tissue. In an attempt to address this concern, a recent study used large-scale proteomics to assess in an unbiased manner the correlation between protein levels in uEVs and kidney tissue from the same animal (142). The quantitative proteomic data identified an overall good positive correlation between uEV and kidney protein abundances, and furthermore provided a catalogue of uEV proteins that track the abundance of the parent protein in the kidney. Although this study supports the reliability of using uEV protein changes to monitor physiological responses and disease mechanisms, the use of a defined cohort of genetically identical animals is a limiting factor and similar large correlations in humans is still lacking. In addition, the usefulness of uEV analysis has to be taken in context of the numerous confounders that can affect results. Many factors, including glomerular filtration rate (GFR), tubular metabolism and reabsorption, dietary consumption, medical conditions, medications, gender and age, can affect an individual’s urine production and composition. In addition as described, multiple steps involved in the pre-analytical phase and isolation when studying uEVs also influence the results of subsequent downstream analyses, such as characterisation and assays to determine EV contents and function.

## The putative “renal-K^+^ switch”

**Figure 3:**
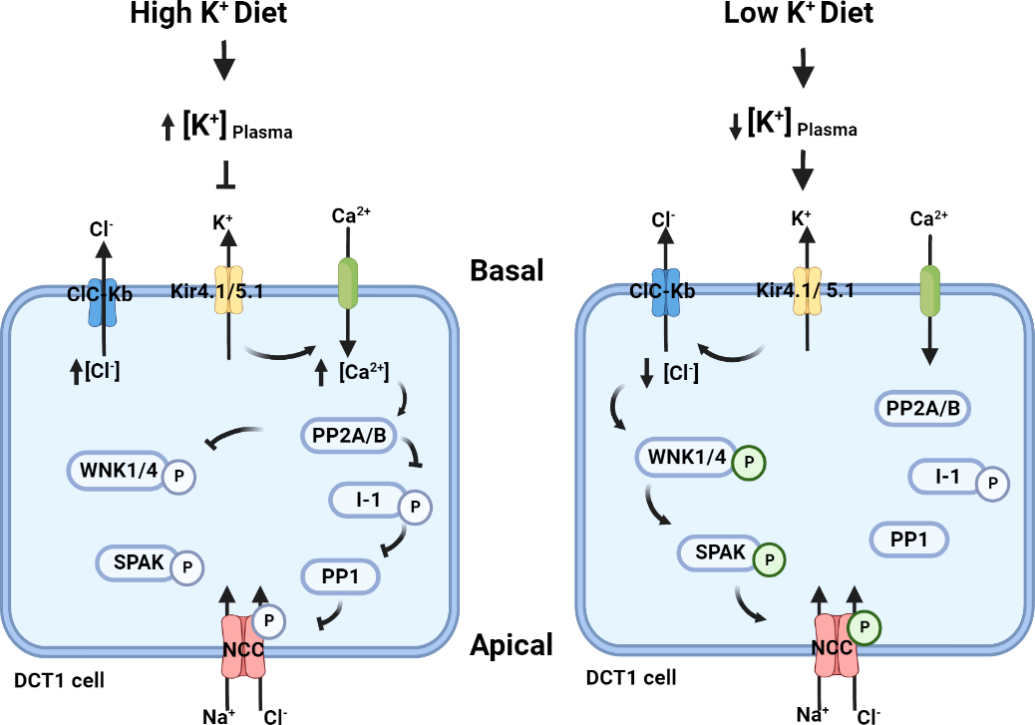
The putative “renal^-^K^+^ switch” mechanism. In the basolateral membrane, K^+^ channels Kir4.1/5.1 and a Cl^-^ channel ClC-Kb can indirectly regulate NCC activity by modifying intracellular [Cl^-^] and hence the autophosphorylation of WNKs (145). High dietary K^+^ intake increases plasma [K^+^], resulting in reduced K^+^ extrusion by Kir4.1 and plasma membrane depolarisation of the early DCT (DCT1). This limits Cl^-^ removal by ClC-Kb and hence intracellular Cl^-^ mediated inhibition of the WNK-SPAK-NCC pathway remains (146–148). In addition, the effects on Kir4.1 may facilitate extracellular Ca2^+^ influx across the membrane via an unknown voltage-gated Ca2^+^ channel. This increased intracellular Ca2^+^ is proposed to activate protein phosphatase 2 (PP2A/B) to inhibit WNK (149, 150), and potentially PP2A/B may modulate the protein phosphatase 1 inhibitor (I-1) protein phosphatase 1 (PP1) pathway leading to NCC dephosphorylation (151–153). In contrast, a low K^+^ diet reduces the plasma K^+^ concentration. Low extracellular K^+^ results in cellular K^+^ extrusion by Kir4.1 leading to membrane hyperpolarization and release of Cl^-^ from the cell through ClC-Kb. The subsequent reduction in intracellular Cl^-^ relieves the inhibition of WNK4 autophosphorylation and allows the WNK-SPAK pathway to phosphorylate and activate NCC, leading to more NaCl reabsorption.

The distal tubule is critical in fine-tuning Na^+^ reabsorption and K^+^ excretion, and these processes are intimately related, as exemplified by amiloride induced hyperkalaemia and the presence of hypokalaemia in patients with thiazide induced hyponatraemia. The primary channels involved in distal tubular Na^+^ handling include ENaC expressed from the late distal convoluted tubule (DCT) through the connecting tubule (CNT) and the medullary collecting duct (CD), and NCC expressed only in the DCT. ENaC is thought to be the major target of aldosterone in the distal nephron. Although NCC activity was originally proposed to be increased by aldosterone, more recent studies indicate this largely occurs through low K^+^ induced increases in NCC phosphorylation (which activates the transporter) *via* activation of the Cl^‐^ -sensitive With-No-Lysine kinases (WNK) and SPS1-related proline/alanine-rich kinase (SPAK) network (11, 13, 143, 144) – a mechanism often referred to as the “renal-K^+^ switch” (**Figure 3**). The switch proposes that a low K^+^ intake acts as a trigger that links K^+^ and NCC regulation. The switch “turns on” NCC in response to a low dietary K^+^ intake, and “turns off” NCC in response to a high K^+^ intake, altering the amount of Na^+^ delivered to downstream ENaC and altering the degree of Na^+^/K^+^ exchange to modulate extracellular fluid (ECF) K^+^ concentrations (13, 144). Hence, although aldosterone stimulates ENaC to promote distal Na^+^ reabsorption and K^+^ excretion, the ECF K^+^ concentration itself regulates NCC abundance and phosphorylation. The switch pathway centers around the WNK-SPAK kinase network (11). Importantly, mutations of WNK1 and WNK4 or components of WNK regulatory ubiquitin ligase complex culin3 (CUL3) and kelch-like family member 3 (KLHL3) cause familial hyperkalaemic hypertension (FHHt, also known as Gordon’s syndrome) by increasing NCC activity. Inhibition of NCC by thiazide diuretics corrects the hypertension and hyperkalaemia in FHHt. These observations, and the use of thiazide diuretics as a BP lowering agent for hypertension, highlight the critical role of NCC in regulation of Na^+^ and K^+^ balance and BP. Unfortunately, difficulties in obtaining human kidney tissue have hampered studies of the “renal-K^+^ switch” model in the clinical setting. To navigate this roadblock, analysis of human uEVs has been used recently as an indirect readout to assess the roles of K^+^ and aldosterone in regulating NCC expression and activity.

## Use of uEVs for studying NCC

The presence of NCC in low-density membrane fractions from urine of normal rats first raised the possibility that analysis of urine samples may be useful in clinical settings (54). By utilising uEVs, Mayan and colleagues demonstrated a marked increase in NCC in FHHt subjects compared to controls, providing evidence of a central role of NCC in the pathophysiology of FHHt (132). The calcineurin inhibitors cyclosporine-A and tacrolimus, often used as anti-rejection drugs in transplant patients, can cause hypertension and hyperkalaemia, resembling FHHt. Calcineurin inhibitor treatment also increased NCC and phosphorylated NCC (pNCC) in human uEVs (154), concomitant with thiazides lowering the blood pressure in these patients (155). In Gitelman syndrome, NCC is absent or reduced in uEVs, consistent with the patients’ renal tissue (156). NCC in uEVs can also differentiate salt-sensitive and salt-resistant hypertensive patients (157). However, no reduction in NCC after high NaCl diet in salt-sensitive hypertensives while fractional sodium reabsorption significantly decreased, and the lack of association between changes in BP with NaCl intake and change in NCC in uEVs in the study suggests that the effect of NaCl intake on NCC may be altered renal sodium handling in salt-sensitive hypertension.

Primary aldosteronism is a common and potentially curable form of hypertension, characterized by excessive production of aldosterone by the adrenal glands that is partially or completely autonomous of the RAAS, and associated with suppressed renin activity and variable but not universal hypokalaemia. In patients with primary aldosteronism, pNCC in uEVs was higher than in patients with essential hypertension, a study which first utilized uEV pNCC as a clinical biomarker (135). Our group examined uEVs from patients with primary aldosteronism undergoing 4-day co-administration of fludrocortisone acetate and oral NaCl loading (fludrocortisone suppression testing, as a means of confirming or excluding primary aldosteronism), with variable amounts of KCl supplements to correct or prevent hypokalaemia during testing (136). There were marked increases in uEV levels of NCC and pNCC at the end of testing, providing evidence that NCC is mineralocorticoid-sensitive. However, an inverse correlation between abundances of NCC and pNCC and plasma K^+^ in the study suggested that the observed effect on NCC may be attributable to changes in plasma K^+^ rather than direct mineralocorticoid stimulation. Soon after, in another group of patients who underwent the same procedure but with a greater rise in plasma K^+^ due to KCl supplementation, the increase in uEV NCC was not apparent (139). This observation is consistent with recent mice studies that demonstrated that K^+^ supplementation reduced the degree of upregulation of NCC abundance induced by aldosterone infusion (158, 159). These observations raise the possibility that higher plasma K^+^ induced by KCl supplements during the testing may counterbalance mineralocorticoid-induced NCC stimulation. However, interpretation of the findings of the two uEV studies was complicated by the fact that they involved mineralocorticoid, KCl and NaCl supplementation. The effect of NaCl loading and volume expansion on the uEV NCC profile and a possible role (if any) of intravenous NaCl loading alone on NCC in patients with primary aldosteronism was recently examined (137). Despite a significant fall in plasma aldosterone in the patients, there were no changes in uEV NCC and pNCC after normalisation, suggesting aldosterone is not a major regulator of NCC abundance and phosphorylation. By contrast, the inverse association of plasma K^+^ with uEV NCC further highlights that ECF K^+^ concentration is a potent regulator of NCC.

In Cushing’s syndrome, NCC and pNCC are more abundant in uEVs of patients with a suppressed RAAS than those with non-suppressed RAAS and healthy controls (140). In this study, patients with Cushing’s syndrome and suppressed RAAS had similar blood pressure but significantly lower plasma K^+^ (although within the normal range) than those with non-suppressed RAAS, with inverse correlations of plasma K^+^ and uEV abundances of NCC and pNCC. These observations are similar to patients with primary aldosteronism, that uEV levels of pNCC are indeed a reliable readout for NCC activity and that plasma K^+^ may override any stimulatory effect of mineralocorticoid/aldosterone on NCC. The hypokalaemia in patients with primary aldosteronism or Cushing’s syndrome may contribute to hypertension through increased Na^+^ reabsorption *via* enhanced NCC activity. Recently, Kong et al. reported that differences in NCC and pNCC in uEVs can be used as biomarker for subtyping of primary aldosteronism and potentially for avoiding the need for adrenal venous sampling (AVS) in differentiating unilateral from bilateral forms (138). The use of uEVs as biomarkers for primary aldosteronism subtyping appears valid; however, uEVs cannot be used to avoid AVS because even if associated to unilateral forms, the side of lateralization cannot be identified. The study concluded that the observed differences were due to higher aldosterone in the unilateral group, but an effect of low K^+^ on NCC and pNCC may have been overlooked because they corrected hypokalaemia with KCl supplements.

## Future perspectives

Use of uEVs as a tool to assess NCC abundance and activity in a variety of human studies has provided insight to the relative roles of K^+^ and mineralocorticoids in NCC regulation. The observations in uEVs support the notion that suppressive effects of K^+^ override stimulatory effects of mineralocorticoid on NCC in humans. Using uEVs to explore molecular mechanisms in humans with the intent to combine them with information from animal models and clinical assessment provides novel insights into the interactions between kidney Na and K handling, blood pressure and the RAAS. However, use of uEV NCC, pNCC or other molecules as biomarkers for diagnosis, prognosis and guidance for treatment is still methodologically challenging. Apart from the already known great differences in uEVs between patients in different gender (160, 161), age (162) and states of disease (131, 163, 164), limiting technical variations throughout the uEV analysis workflow and establishing standardised protocols are required before clinical application of uEV assessment. Moreover, the lack of robust molecular normalisation controls or normalisation controls for urine volume is still a major challenge in the field (165). Although the International Society of Extracellular Vesicles has provided recommendations for uEV research (107), new information derived from research in this rapidly growing field has evolved so that they are not universally accepted among different researchers. Nevertheless, with ongoing refinement and standardization, the study of uEVs remains a promising tool, for both research into disorders of BP regulation and possibly for incorporation into clinical practice.

## Author contributions

AW drafted the paper and made the figures and tables. MJW, RAF and MS revised the paper. All authors contributed to the article and approved the submitted version.

## Funding

This work is supported by a grant from the Leducq Foundation (Potassium in Hypertension Network 17CVD05).

## Acknowledgments

Cartoons in Figures were created with BioRender.com.

## Conflict of interest

The authors declare that the research was conducted in the absence of any commercial or financial relationships that could be construed as a potential conflict of interest.

## Publisher’s note

All claims expressed in this article are solely those of the authors and do not necessarily represent those of their affiliated organizations, or those of the publisher, the editors and the reviewers. Any product that may be evaluated in this article, or claim that may be made by its manufacturer, is not guaranteed or endorsed by the publisher.
